# Media Image Landscape: Age Representation in Online Images

**DOI:** 10.1093/geroni/igaa057.332

**Published:** 2020-12-16

**Authors:** Colette Thayer, Laura Skufca

**Affiliations:** AARP, Washington, District of Columbia, United States

## Abstract

This study looked at the extent to which the 50-plus population is portrayed in media images online. A random sample of images was drawn from 2.7 million images downloaded from professional and semiprofessional domains and social distributions for brands and thought leaders. Natural language processing technology was employed to find images using topical guides chosen to be reflective of online images. Results of this study showed that while some media has moved toward more positive visual representation of older people, the 50-plus population is still not accurately portrayed in the media. For example, while nearly half of the U.S. adult population is age 50-plus, only 15% of images containing adults include people this age. In addition, when the 50-plus are shown, they are more likely to be portrayed negatively than those under age 50. The 50-plus population is often portrayed as dependent and disconnected from the rest of world although most are actively engaged in their communities. They are rarely shown with technology and in work settings. Furthermore, while a myriad of vibrant personalities come across in images of adults under age 50, the representation of people 50-plus starts to homogenize and exaggerate stereotypical and outdated physical appearance characteristics. This study demonstrates the need for visual representations that reflect greater diversity and authenticity of the 50-plus population as these images affect the attitudes, expectations, and behaviors of older and younger people alike. Keywords: ageism, reframing aging, media image representation

